# Fine Mapping of *Lr49* Using 90K SNP Chip Array and Flow-Sorted Chromosome Sequencing in Wheat

**DOI:** 10.3389/fpls.2019.01787

**Published:** 2020-02-04

**Authors:** Vallence Nsabiyera, Deepak Baranwal, Naeela Qureshi, Pippa Kay, Kerrie Forrest, Miroslav Valárik, Jaroslav Doležel, Matthew J. Hayden, Harbans S. Bariana, Urmil K. Bansal

**Affiliations:** ^1^ Faculty of Science, School of Life Sciences and Environment, The University of Sydney Plant Breeding Institute, Cobbitty, NSW, Australia; ^2^ Agriculture Victoria Research, AgriBio, Bundoora, VIC, Australia; ^3^ Institute of Experimental Botany of the Czech Academy of Sciences, Centre of the Region Haná for Biotechnological and Agricultural Research, Olomouc, Czechia; ^4^ School of Applied Systems Biology, La Trobe University, Bundoora, VIC, Australia

**Keywords:** adult plant resistance, chromosome sorting, Infinium iSelect 90K SNP array, leaf rust, marker assisted breeding

## Abstract

Leaf rust, caused by *Puccinia triticina*, threatens global wheat production due to the constant evolution of virulent pathotypes that defeat commercially deployed all stage-resistance (ASR) genes in modern cultivars. Hence, the deployment of combinations of adult plant resistance (APR) and ASR genes in new wheat cultivars is desirable. Adult plant resistance gene *Lr49* was previously mapped on the long arm of chromosome 4B of cultivar VL404 and flanked by microsatellite markers *barc163* (8.1 cM) and *wmc349* (10.1 cM), neither of which was sufficiently closely linked for efficient marker assisted selection. This study used high-density SNP genotyping and flow sorted chromosome sequencing to fine-map the *Lr49* locus as a starting point to develop a diagnostic marker for use in breeding and to clone this gene. Marker *sunKASP_21* was mapped 0.4 cM proximal to *Lr49*, whereas a group of markers including *sunKASP_24* were placed 0.6 cM distal to this gene. Testing of the linked markers on 75 Australian and 90 European cultivars with diverse genetic backgrounds showed that *sunKASP*_*21* was most strongly associated with *Lr49*. Our results also show that the *Lr49* genomic region contains structural variation relative to the reference stock Chinese Spring, possibly an inverted genomic duplication, which introduces a new set of challenges for the *Lr49* cloning.

## Highlights

High-density SNP genotyping and flow-sorted chromosome sequencing were used to fine map adult plant leaf rust resistance gene *Lr49*.

## Introduction

Leaf rust, caused by *Puccinia triticina* (Pt), is one of the most important diseases of wheat worldwide and can result in yield losses of up to 70% (Kolmer, 2005). While wheat has inherent defense mechanisms to resist diseases, an emphasis on selection for high yield and other desirable traits has resulted in a narrow genetic base for disease resistance (Borlaug, 2007). The release of resistant cultivars is the best strategy to control leaf rust, and to reduce production costs and risk of environmental pollution resulting from fungicide usage (Bariana, 2003; Bariana et al., 2007; Bariana and Bansal, 2017).

Many leaf rust resistance genes have been identified and named in wheat (McIntosh et al., 1995; McIntosh et al., 2013; Bariana and Bansal, 2017). Resistance genes include two categories; all stage resistance (ASR) and adult plant resistance (APR). The ASR genes are effective throughout the life of the plant, whereas adult plant resistance genes are effective only at adult plant stage. A majority of the formally named genes confer ASR (McIntosh et al., 1995; McIntosh et al., 2013). ASR genes exhibit hypersensitive reaction to condition a high level of resistance against avirulent pathogen isolates. However, they are prone to breakdown when the pathogen evolves to acquire virulence. The ASR genes can be identified at the seedling stage under greenhouse conditions. In contrast, APR genes express at the postseedling stages and retard pathogen growth. They are considered durable due to their race nonspecific nature. Examples of pleiotropic APR genes include *Lr34/Yr18* (Singh, 1992), *Lr46/Yr29* (Singh et al., 1998), *Lr67/Yr46/Sr55* (Hiebert et al., 2010; Herrera-Fossel et al., 2011), *Lr68* (Herrera-Foessel et al., 2012) and *Lr75* (Singla et al., 2017). Some APR genes show race-specific responses, and these include *Lr12* (McIntosh et al., 1995) and *Lr22b* (Dyck, 1979). While *Lr48* and *Lr49* were assigned by Saini et al. (2002) to the hypersensitive category based on monocyclic flag leaf tests, Bariana and Bansal (unpublished results) observed these genes to be slow rusting under polycyclic infection conditions in the field.

Traditionally, the development of new wheat cultivars has followed conventional phenotypic selection of desirable traits. Although this approach remains effective, it faces significant challenges due to the length of time taken to release a new cultivar (Forster et al., 2015). Recent advances in wheat genomics have led to the development of more efficient and precise approaches for wheat improvement (Dubcovsky and Dvorak, 2007; Bariana et al., 2013). For example, the identification of DNA markers linked with rust resistance genes has largely overcome the limitations of phenotypic selection for pyramiding two or more genes in breeding programs (Bariana et al., 2007; Bariana and Bansal, 2017). Similarly, the availability of high-density genotyping platforms such as DArTseq (Diversity Arrays Technology, Bruce, Australia; Cruz et al., 2013) and Infinium iSelect 90K SNP bead chip array (Wang et al., 2014) have expedited the mapping of economic traits (Bariana and Bansal, 2017). The development of simple gel-free marker genotyping systems such as kompetitive allele-specific PCR (KASP; LGC Genomics, UK) have encouraged marker assisted selection in breeding programs.

The rate for development of trait-linked DNA markers has also been accelerated by the increasing availability of genomic resources and tools supporting high throughput genomics. For example, methods have been developed to isolate specific chromosomes using flow cytometry (Vrána et al., 2000; Giorgi et al., 2013), which can then be sequenced to interrogate individual chromosome DNA code. Such approaches are particularly useful in polyploid species such as wheat, because they not only reduce the genome complexity to a single chromosome but also eliminate problems associated with presence of homoeologous genomes for sequence assembly (Doležel et al., 2007). The ability to sequence individual chromosomes adds a new dimension to marker development and gene cloning in allopolyploids including wheat (Doležel et al., 2012; International Wheat Genome Sequencing Consortium (IWGSC), 2014).

An Indian cultivar VL404 (Kentana/Bungulla//Frontana/General-Urquiza/3/ST464/PI-74106) was released in 1973 by Vivekananda Parvatiya Krishi Anusandhan Sansthan, Almora. This cultivar was susceptible at the two-leaf stage but showed resistance at the flag-leaf stage against Indian Pt pathotypes in monocyclic inoculations and the underlying resistance locus was formally named *Lr49* (Saini et al., 2002). *Lr49* was mapped on the long arm of chromosome 4B (Bansal et al., 2008) using the VL404/WL711 RIL population, however, the flanking markers were not sufficiently close for efficient marker assisted selection. The aim of this study was to fine map the gene *Lr49* using recently published assembled reference genome sequence for variety Chinese Spring (Appels et al., 2018) and flow sorted chromosome 4B sequence for VL404 and WL711. This is a first step towards cloning the causal gene for *Lr49* and developing a diagnostic marker for use in marker assisted selection.

## Materials and Methods

### Plant and Pathogen Materials

The VL404/WL711 F_6:8_ derived recombinant inbred line (RIL) population used in this study comprised 181 lines. Pedigree information for both parents is described in the earlier study (Saini et al., 2002). A diverse set of Australian (75) and European (90) wheat cultivars was used to test the strength of linkage between markers developed in this study and *Lr49*. DNA was extracted from each wheat line using the method described in Bansal et al. (2014a).

### Greenhouse Tests

Eight to 10 seeds of each RIL and parents were sown in 9-cm diameter pots as four lines per pot. Twenty grams of complete fertilizer Aquasol dissolved in 10L of tap water was applied to pots filled with potting mix before sowing. Plants were grown to the 4^th^ leaf stage at 20°C in a rust-free microclimate room prior to inoculation. Urea was applied every week prior to inoculation with *Lr49*-avirulent Pt pathotype 76-1,3,5,10,12 (culture no 539). The inoculation procedure described in Bansal et al. (2008) was followed. Rust response assessments were made 18 to 20 days post-inoculation using the infection type (IT) scale detailed in McIntosh et al. (1995). Briefly 0-4 infection type scale was used and RILs classified <3 were considered resistant and >3 susceptible.

### Sorting and Sequencing of Chromosome 4B

Suspensions of intact mitotic chromosomes were prepared from synchronised root meristems of parental lines VL404 and WL711 (Vrána et al., 2000; Vrána et al., 2012) and GAA microsatellites on the chromosomes were labeled in suspension by fluorescein isothiocyanate (FITC) using the protocol for Fluorescence In Situ Hybridization In Suspension (FISHIS) (Giorgi et al., 2013). Genomic DNA was stained by 4’,6-diamidino2-phenylindole (DAPI) and chromosomes were analyzed on a FACSAria II SORP high speed flow sorter (BD Biosciences, San José, USA) as described in Kubaláková et al. (2002). One thousand chromosomes from each cluster were sorted onto a microscopic slide into a drop of 10 µl of PRINS buffer supplemented with 5% sucrose (Kubaláková et al., 1997). To assign chromosomes to an individual cluster on a dot plot, FISH with probes for GAA and Afa repeats was used to identify flow-sorted chromosomes and to assess purity. Chromosomal DNA of 4B was amplified by the Multiple Displacement Amplified (MDA) approach using the Illustra GenomiPhi V2 DNA amplification kit (GE Healthcare, http://www.gehealthcare.com) as described in Šimková et al. (2008). Sequencing libraries were generated using the Nextera DNA sample preparation kit (Illumina Inc, San Diego, CA, USA) and 50 ng of DNA was amplified (according to the manufacturer’s instructions, with the exception for usage of 3 μl of TDE1 for DNA fragmentation). Libraries with insert sizes of 600–800 bp were selected for sequencing. The insert sizes were verified using the Agilent DNA 1000 Kit (Agilent Technologies, Inc.) and concentrations were assessed by the KAPA Library Quantification Kit (Kapa Biosystems, Woburn, USA). The libraries were sequenced as paired-end reads using the HiSeq Rapid SBS Kit v2 (2x250 bp) (Illumina Inc, San Diego, CA, USA).

### SNP Detection Using Flow Sorted Chromosome 4B Sequence

GYDLE software (Gydle Inc. Bioinformatics Service, Quebec City, Canada; http://www.gydle.com) was used to quality filter the raw sequence reads (minimum phred score 20; minimum read length 50 bp) derived from flow-sorted 4B chromosome sequences of VL404 and WL711 and to align the filtered reads to the International Wheat Genome Sequencing Consortium (IWGSC) reference genome sequence assembly for cultivar Chinese Spring (RefSeq assembly v1.0; Appels et al., 2018). Gydle software performs an exhaustive alignment search to guarantee each paired-end read is aligned at its best mapping position, providing the alignment score that exceeds 80% sequence homology. Paired-end reads that align to multiple positions with equal scores are randomly distributed across those positions to ensure that all alignment positions are fully and correctly reflected. The SNP variant discovery and genotype calling was performed using the aligned paired-end sequence reads for WL711 and VL404 and GYDLE “findsnp” function.

### DNA Genotyping

Microsatellite markers previously reported to be linked with *Lr49* (Bansal et al., 2008) were genotyped on the entire RIL population and parents following the amplification conditions described in Bansal et al. (2014b). Infinium iSelect 90K SNP genotyping was performed on 12 resistant and 12 susceptible RILs, as reported in Wang et al. (2014). Genotype calling was performed using GenomeStudio (Illumina) and a custom perl script to assign genotype calls. Closely linked 90K SNPs and those identified from the flow-sorted chromosome sequences were converted into kompetitive allele-specific PCR (KASP) assays (LGC Genomics) following the manufacturers guidelines. For each KASP marker, two allele-specific forward primers and one common reverse primer were designed using BatchPrimer3 v1.0 (https://wheat.pw.usda.gov/demos/BatchPrimer3/) software. The PCR reaction contained 3 µl of DNA (30ng/µl), 5 µl KASP mix (LGC Biosearch Technologies), and 0.11 µl of primer mix (12 µM of each allele specific primer and 30 µM of reverse primer). Reaction was performed in CFX96 real time PCR machine (Biorad, USA) with the following cycling conditions: 15 min at 94°C; 10 touchdown cycles of 20 s at 94°C, 60 s at 65–57°C (dropping 0.8°C per cycle); and 26–35 cycles of 20 s at 94°C, 60 s at 57°C. Flourescence reading was taken at 40°C for 30 s and were analysed using allelic discrimination function. The KASP markers derived from 90K SNPs were named with the prefix KASP, followed by a number corresponding to the SNP index on the Infinium bead chip. The KASP markers derived from flow-sorted chromosome sequence variants were designated by the prefix *sunKASP* (sun = Sydney University) followed by a consecutive number.

### High Resolution Mapping

VL404 was crossed with Avocet S to develop a high-resolution mapping population consisting of 2560 F_2_ plants. DNA was extracted from each F_2_ plant and tested with the *Lr49* flanking markers. Plants showing recombination between the flanking markers were transplanted and the high resolution F_3_ family was generated and phenotyped with Pt pathotype 76-1,3,5,10,12 at the 4^th^ leaf stage.

### Data Analyses and Genetic Mapping

The RIL population was categorized as homozygous resistant (HR) or homozygous susceptible (HS) based on the phenotypic scores of parents. Chi-squared (χ^2^) test was used to determine the goodness of fit of the observed segregation to the expected genetic ratios. Alleles for SNP and SSR markers were scored as A and B for parents VL404 and WL711, respectively.

A genetic map was generated using MapManager Version QTXb20 (Manly et al., 2001) and the Kosambi map function (Kosambi, 1943). The linkage map was drawn according to Voorrips (2002). A likelihood of odds (LOD) score of 3.0 was used as the threshold for declaring linkage among loci.

The genetic-physical map viewer Pretzel (Keeble-Gagnère et al., 2019) was used to identify and visualize structural variation in the genomic region containing the *Lr49* locus.

## Results

### Genetic Analysis

VL404 (*Lr49*) and WL711 exhibited infection type (IT) X (mesothetic response which includes more than two infection types on the same leaf) and IT3+, respectively, when inoculated with Pt pathotype 76-1,3,5,10,12 at the 4^th^ leaf stage under greenhouse conditions. Leaf rust test on VL404/WL711 RIL population revealed clear segregation of *Lr49* (99 resistant: 82 susceptible; χ²_1:1_ = 1.59, nonsignificant at *P = 0.05* and *1d.f.*).

### Chromosome Sorting and Sequencing

Thirty-five thousand copies of chromosome 4B from each of VL404 and WL711 were sorted with 97% and 98% purity, respectively, and amplified by MDA. To minimize the risk of representation bias, the products from three independent MDA reactions were pooled. The amplification and pooling of 4B chromosomal DNA from VL404 and WL711 yielded 7.88 and 8.36 µg DNA, respectively, and paired-end sequencing provided 70,519,221 and 70,685,036 reads. Following quality filtering, the filtered reads were aligned to the reference genome sequence assembly of cultivar Chinese Spring. This resulted in 68.8 and 64.7% of the filtered reads uniquely mapping to chromosome 4B, representing 16.5 and 17.3 fold coverage for VL404 and WL711, respectively.

### Molecular Mapping

**Figure 1:**
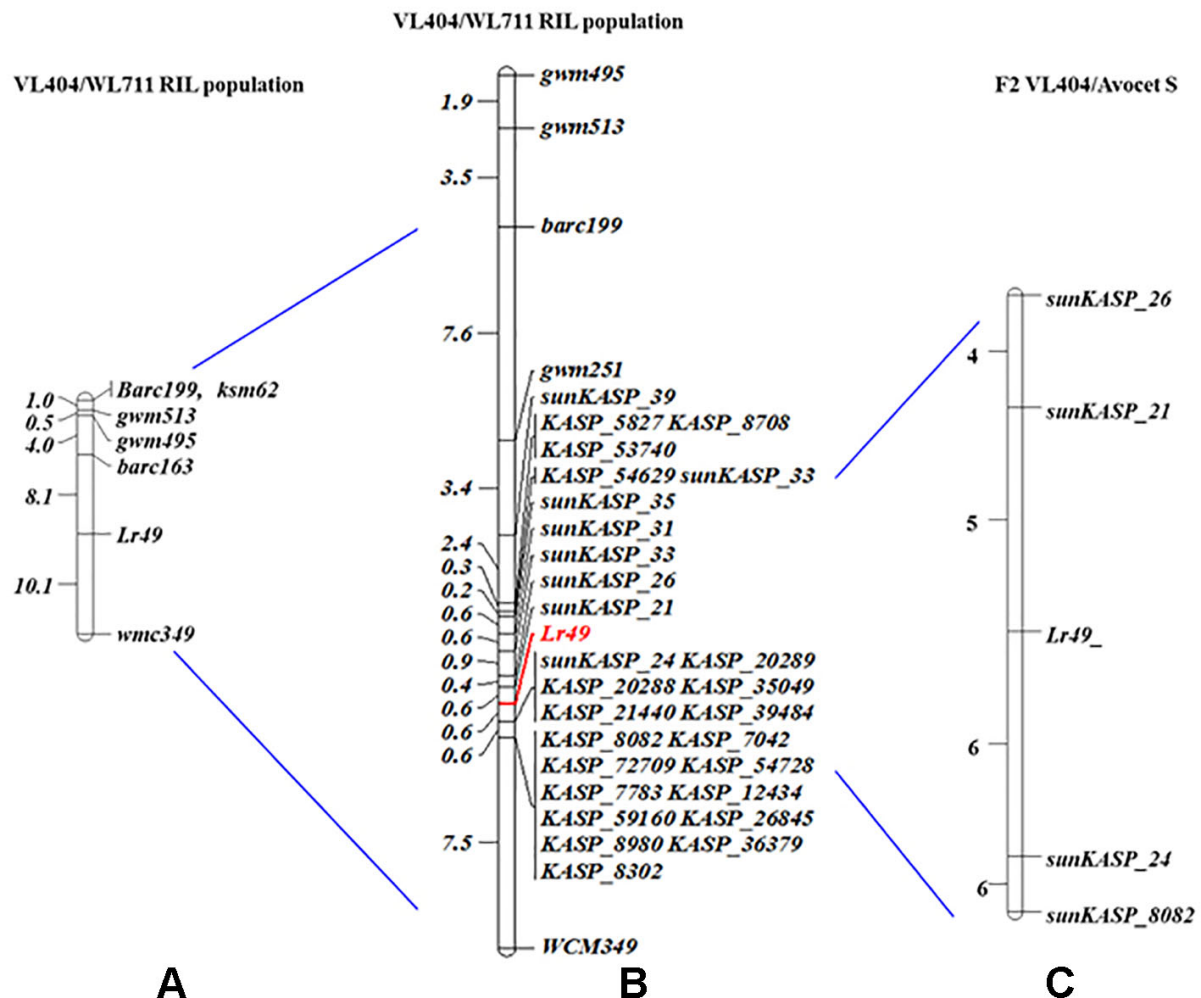
Genetic linkage map of chromosome 4BL for the VL404/WL711 RIL population **(A)** Bansal et al. (2008), **(B)** present study (resistance gene is shown in red text), and **(C)** high resolution map of VL404/Avocet S (number of recombinants is given on the left).

Forty-five SNPs from the iSelect 90K Infinium array showed linkage with *Lr49* and were converted into single-locus KASP assays. Twenty-one KASP markers that clearly discriminated the parents (**Table 1**) were genotyped on the entire RIL population and integrated into the previously reported microsatellite marker-based genetic linkage map carrying *Lr49* (**Figure 1**). The closest proximal marker *KASP_54629* mapped 2.7 cM from *Lr49,* whereas the closest distal markers at 0.6 cM and included several co-segregating KASP markers.

**Table 1:** Primer sequences for kompetitive allele-specific PCR (KASP) markers designed from SNP sequences that showed association with *Lr49* on chromosome 4BL and SNPs discovered from the sqeuences of flow sorted chromosome 4B of parental genotypes.

KASP marker	SNP ID	Forward (Allele 1) primer	Forward (Allele 2) primer	Common/reverse primer
*KASP_5827*	*IWB5827*	gaagcagctggcagcactca	gaagcagctggcagcactcg	gctcagcctcaaggtcggtgtt
*KASP_7042*	*IWB7042*	tccaagttgactcaagagacgaga	ccaagttgactcaagagacgagg	cctcctacgcaacaaccgacacaa
*KASP_7783*	*IWB7783*	agtaagaggcactaccgttcagatt	aagaggcactaccgttcagatc	agagggcgtgctttccaagtgaat
*KASP_8082*	*IWB8082*	ttgctggtctttagaaatccctc	gctttgctggtctttagaaatcccta	cttgcactaacatcacaacccccat
*KASP_8302*	*IWB8302*	acccttttacaacaacttcattcgc	ctacccttttacaacaacttcattcgt	gggaataatcagggattgaccccta
*KASP_8708*	*IWB8708*	ggtcgtggtgacgcacgcaa	gtcgtggtgacgcacgcag	ttcacatgaccatggccaggagat
*KASP_8980*	*IWB8980*	agagcagaattgattgctgcaact	agagcagaattgattgctgcaacc	ctccacttcctcactgtcatctgtt
*KASP_12434*	*IWB12434*	cgcacgacgagtgctgcca	gcacgacgagtgctgccc	gtcaaggtcgaccaacccctgaa
*KASP_20288*	*IWB20288*	gtcctcaatttggagatgcgctt	gtcctcaatttggagatgcgctg	atcaagatgtaattctggattcagcaagaa
*KASP_20289*	*IWB20289*	tcttttatactcacattctgaatcaggat	cttttatactcacattctgaatcaggac	ctccgagagtcaaaatacaagaagtgttt
*KASP_21440*	*IWB21440*	gtccccagctaatcctgtggaa	ccccagctaatcctgtggag	tagtttctgtagcttcggttgatacctt
*KASP_26845*	*IWB26845*	cagtttaatatgcagcagcactta	ctcagtttaatatgcagcagcacttc	gacgcagatccatctcagaaggtaa
*KASP_35049*	*IWB35049*	aagcgaaagagaaactatttacagt	ctaagcgaaagagaaactatttacagc	tttagacacagcgatacgttgtacatgtt
*KASP_36379*	*IWB36379*	gtgaccttatgactggtagaag	cctgtgaccttatgactggtagaaa	aaatcgcaatttsaacatgaattcagtctt
*KASP_39484*	*IWB39484*	agtcaatgcaaggaggagaaa	ctagtcaatgcaaggaggagaag	gccgcttgttaggcttctggctt
*KASP_53740*	*IWB53740*	tgtcatcttcattttcagcatctgca	gtcatcttcattttcagcatctgcg	gatccagaggagaaaggttctgcta
*KASP_54629*	*IWB54629*	gtgtctccaagtgacagttgaatgt	gtctccaagtgacagttgaatgc	atccagcttcttgtacagcggagaa
*KASP_54728*	*IWB54728*	gatgacatcgacggcgaactga	atgacatcgacggcgaactgg	acaccggctggtatgccaccaa
*KASP_59160*	*IWB59160*	aagatttgctttcgatccgtacttca	agatttgctttcgatccgtacttcg	gtaacaacgattcaaatgtggacacgaaa
*KASP_72709*	*IWB72709*	cgatcctaatgaaccgacgtattgt	gatcctaatgaaccgacgtattgc	accgtagctgacttggttgcagaa
*KASP_1109*	*IWB1109*	tattcatcttacgattcttaaatacttccaat	catcttacgattcttaaatacttccaac	ccagggttgtgtgccttcctttatt
*sunKASP_21*	–	gattcgaatgtttttgtaggatttc	ttcagatctaaaatcacggcact	ctattaacgtagagcccagtgc
*sunKASP_24*	–	ttcgattacccgggagc	ttcgattacccgggagt	tgggttaagggcaagaaaca
*sunKASP_26*	–	agtaccaaatgcagcaaaaaaa	cagtaccaaatgcagcaaaaaat	ctttggcccaagttgtgtct
*sunKASP_31*	–	tcaatcatttactttcatgcgag	aatgtaattttattttgttttgcttgc	caccgaccaccattgttcta
*sunKASP_33*	–	catgtcaatagttatgcactcaaattg	aatctttttgctagccttcatctc	tggtccaagtacaggtctacca
*sunKASP_35*	–	caaatcctaaaagccaagatgc	ttcatttcgggactggga	cggagctatttttggaccag
*sunKASP_39*	–	caccatctcctcctcattatca	catcctctagaacaatggtggtc	ctcttcccgttgcaagaaat

Allele 1 primer labeled with FAM: GAAGGTGACCAAGTTCATGCT;

Allele 2 primer labeled with HEX: GAAGGTCGGAGTCAACGGATT

To further increase marker density for the *Lr49* region, SNP discovery was performed using the flow-sorted chromosome sequences for VL404 and WL711. Twenty-eight SNPs with approximately even physical spacing were selected to span the *Lr49* region and converted into KASP assays. Seven of these markers (**Table 1**) produced clear parental clusters and could be unambiguously scored on the RIL population. Integration of these KASP markers into the genetic map for the *Lr49* region, resulted in *sunKASP_21* mapping at 0.4 cM proximal and *sunKASP_24* (clustered with five additional KASP markers derived from 90K SNPs) at 0.6 cM distal to *Lr49* (**Figure 1B**).

### Construction of High-Resolution Map

Markers *sunKASP_21, sunKASP_26, sunKASP_24* and *KASP_8082* that flanked *Lr49* were genotyped on 2560 VL404/Avocet S F_2_ plants to identify recombinants for the construction of a high-resolution map. Twenty-one recombinants were observed. The recombinant F_3_ families were scored as homozygous resistant, homozygous susceptible and segregating. Five and six recombinants were observed between *Lr49* and markers *sunKASP*_*21* and *sunKASP*_*24,* respectively (**Figure 1C**).

### Assessing Marker Linkage Using Unrelated Materials

Flanking markers *sunKASP_21*, *sunKASP_24, KASP_20289, KASP_20288, KASP_35049, KASP_21440* and *KASP_39484* were genotyped on a diverse set of 75 Australian and 90 European cultivars, unlikely to carry *Lr49*, to test the marker linkage with *Lr49*. Across the diverse germplasm, the proximal marker *sunKASP_21* showed the strongest linkage, amplifying the susceptible WL711 (T:T) allele in all cultivars, except Gazelle, Safir and JO 8023, which amplified the resistance VL404 allele (C:C) (**Table 2**). In contrast, the distal markers (*sunKASP_24, KASP_20289, KASP_20288, KASP_35049, KASP_21440* and *KASP_39484*) showed poor linkage, amplifying both the resistant and susceptible alleles (data not shown).

**Table 2:** Validation of closely linked kompetitive allele-specific PCR (KASP) marker on Australian and European wheat cultivars.

Cultivars/lines	*sunKASP*_21
VL404 (*Lr49*)	C:C
WL711	T:T
AGT Katana, Axe, Baxter, Beaufort, Bolac, Calingiri, Carnamah, Catalina, Chara, Cobra, Corack, Correll, Crusader, Dart, Derrimut, Diamondbird, EGA Bonnie Rock, EGA Bounty, EGA Burke, EGA Gregory, EGA Wedgetail, EGA Wylie, Elmore CL PLus, Emu Rock, Envoy, Espada, Estoc, Forrest, Fortune, Gauntlet, GBA Sapphire, Giles, Gladius, Grenade CL Plus, Impala, Impose CL Plus, Janz, Justica CL Plus, King Rock, Kord CL Plus, Kunjin, Lang, Lincoln, Livingston, Mace, MacKellar, Magenta, Merinda, Merlin, Naparoo, Orion, Phantom, Preston, Scout, Sentinel, Shield, Spitfire, SQP Revenue, Strzelecki, Sunco, Sunguard, Suntop, Sunvale, Sunvex, Sunzell, Ventura, Waagan, Wallup, Wedin, Westonia, Wyalkatchem, Wylah, Yandanooka, Yitpi, Young	T:T
Gazelle, JO 8023, Safir	C:C
Apu, Aros, Atson, Avle, Bastian, Bjarne, Blanka, Børsum, Boru, Canon, Dala, Dalarna, Diamant, Diamant ll, Drabant, Dragon, ELS 6404 - 102 - 3, Ergo, Eroica, Extra Kolben, Fagott, Fram l, Fram ll, Fylgia l, Fylgia ll, Haarajärvi ME0102 Apu, Halland, Horsmanaho ME201 Timantti, J-03, Järvenkylä ME0302 Timantti, JO 3524, Jokikylä ME0505 Apu, Kadett, Kärn ll, Kenya Farmer, Kimmo, Kiuru, Kota, Laitiala AP0103, Landvårkveite, Lantvete från Dalarna, Lantvete från Halland, Lavett, Manu, Monola ME1301, Møystad, MS273-150, Naxos, Nemares, Nora, Norrøna, østby, Polkka, Pompe, Pondus, Prins, Progress, Rang, Reno, Ring, Rival, Rollo, Rubin, Runar, Ruso, Saffran, Sappo, Sibirian, Skirne, Snøgg II, Snøgg l, Sober, Sopu, Sport, Svenno, Timantti, Timantti Paavo, Tjalve, Touko, Troll, Trym, Ulla, Vinjett, Vitus, Walter, William, WW 20299, Zebra,	T:T

### Identification Closely Linked Markers

**Figure 2:**
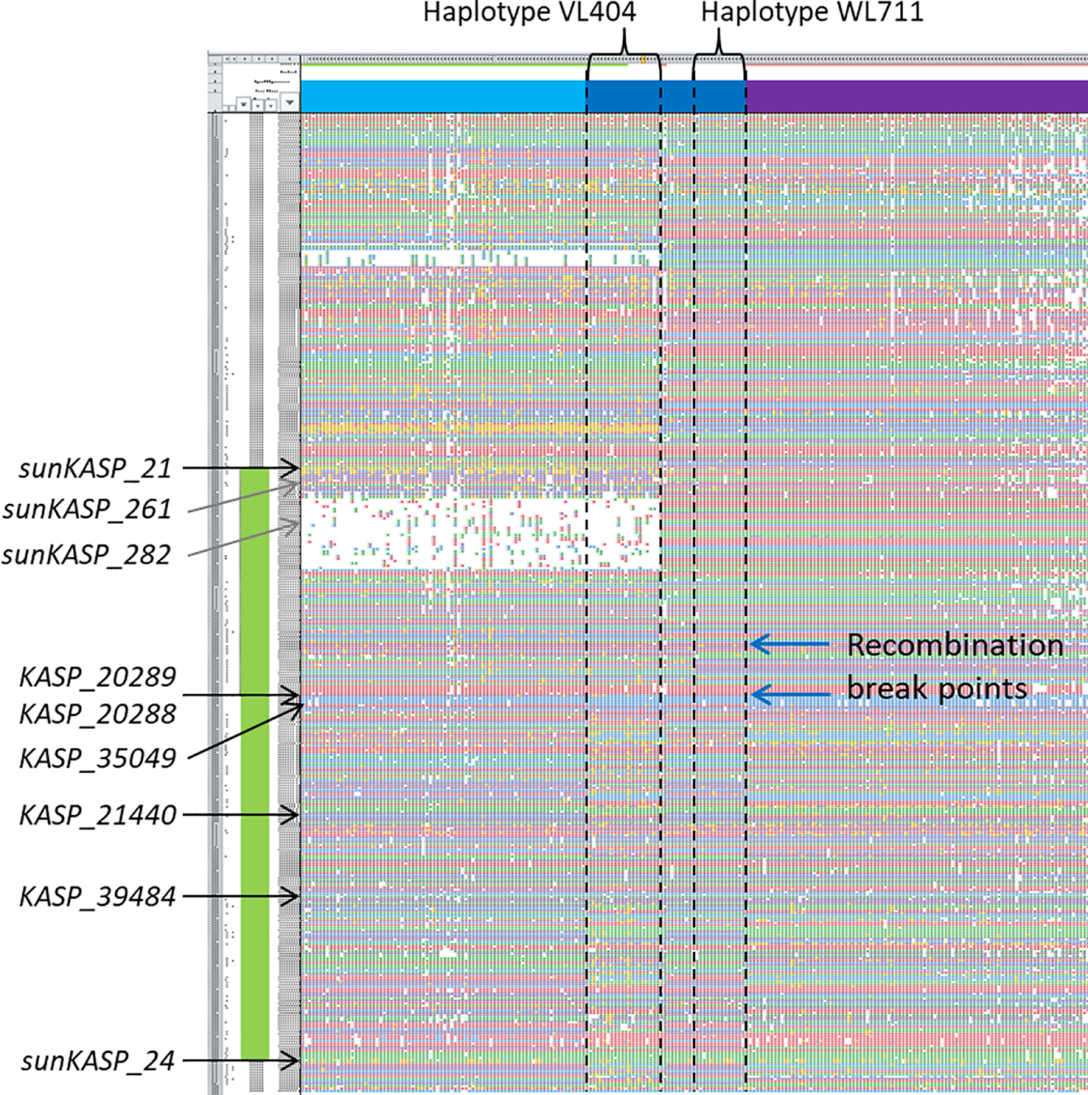
Exome SNP genotypes for 890 globally diverse accessions across the *Lr49* region (green box). The *sunKASP_21* maps proximal to *Lr49*, while *KASP_20289, KASP_20288, KASP_35049, KASP_21440, KASP_39484,* and *sunKASP_24* cosegregate and map distally to *Lr49* in the RIL mapping population. Physical mapping positions for the flanking markers are indicated by black arrows. Two recombination breakpoints present between *sunKASP_21* and *KASP_20289* are shown by blue arrows. Two additional markers, *sunKASP_261* and *sunKASP_282*, developed from parental SNPs proximal to the recombination break points and mapped in the RIL population were not linked to *Lr49*. The physical mapping position for these two markers is denoted by grey arrows.

Exome SNPs from 890 globally diverse accessions (He et al., 2019) located within the *Lr49* region were used to identify recombination hot spots distal to marker *sunKASP_21*. The physical order of SNPs across the *Lr49* region were used to identify five major haplotypes. VL404 belonged to one of these haplotypes and WL711 to another. Across the five haplotypes, two recombination sites were observed distal to the physical mapping position of *sunKASP_21* and proximal to those of the co-segregating markers (*sunKASP_24, KASP_20289, KASP_20288, KASP_35049, KASP_21440* and *KASP_39484*) in the RIL population (**Figure 2**). The recombination sites explain the breakdown in linkage (many false positives) observed in the diverse panel of Australian and European cultivars for markers distal to *Lr49*.

To develop markers between the two recombination sites and *sunKASP_21*, 35 exome SNPs proximal to the recombination sites and distal to *sunKASP_21* were converted into KASP markers. However, only two of the KASP markers (*sunKASP_261* and *sunKASP_282*) were polymorphic between VL404 and WL711 and produced a scorable pattern. When genotyped on the RIL population and integrated into the genetic map, neither marker was closely linked to *Lr49*. Further, based on the physical position of *sunKASP_282* in the reference genome assembly of cultivar Chinese Spring, the marker fell within a putative deletion in the haplotype corresponding to VL404 (**Figure 2**). No additional SNP from the parental flow-sorted chromosome sequences could be identified for the region between *sunKASP_21* and the two recombination sites.

### Genomic Structure of the *Lr49* Region

**Figure 3:**
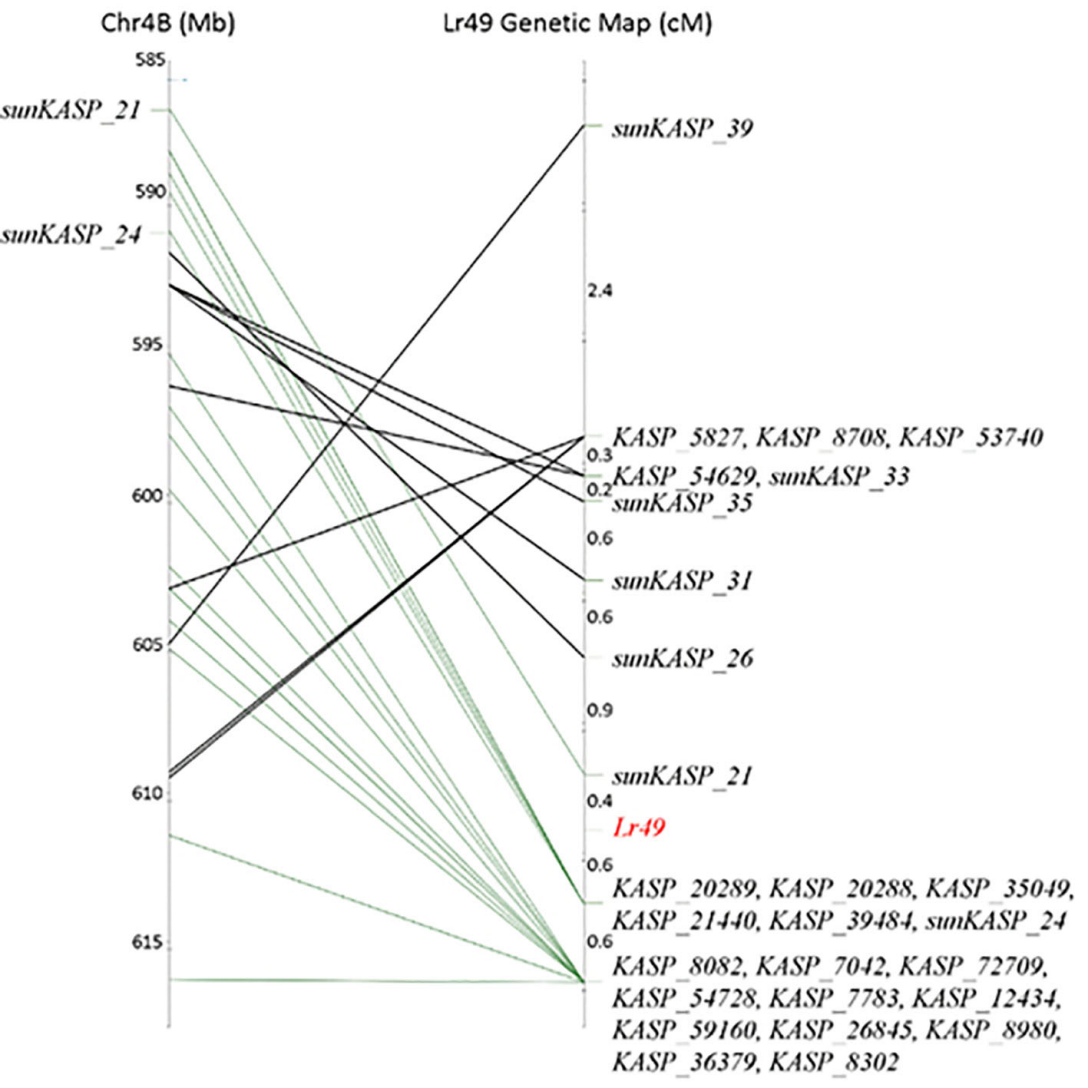
Comparison of *Lr49* genetic linkage map with the 585 to 617 Mbp interval of Chinese Spring chromosome 4B physical map using Pretzel, a genetic map viewing software (http://plantinformatics.io). Markers distal to *sunKASP*_26 are colinear with the physical map (green connections). The genetic map order of markers proximal to *sunKASP*_21 does not reflect the physical map order in Chinese Spring (black connections).

Comparison of marker loci order in the genetic linkage map for the VL404/WL711 RIL population with the physical positions of the same marker loci in the reference genome assembly for Chinese Spring revealed a complex pattern (**Figure 3**). It showed that the marker loci order distal to *sunKASP_21* in the RIL population was colinear with Chinese Spring, while the marker loci order proximal to *sunKASP_26* was inverted. The inversion was contained within the genomic region of Chinese Spring that appeared to be colinear with the RIL population distal to marker *sunKASP_26*. As repeated genotyping of the RIL population with the KASP markers produced the same result, the complex pattern suggested the presence of structural variation between one or both parents of the RIL population and Chinese Spring. Comparison of the physical map order of high confidence genes across the orthologous region in *Ae. tauschii* chromosome 4D, and homoeologous regions in Chinese Spring chromosome 4D (IWGSCv1.0), emmer chromosome 4B (Avni et al., 2017) and Chinese Spring chromosome 4B (IWGSCv1.0) showed no evidence for structural rearrangements (**Supplementary Figure 1**), indicating that the structural variation was present in one or both parents and not Chinese Spring.

The genomic region between *sunKASP_21* and the markers cosegregating with *sunKASP_24* in the Chinese Spring reference genome assembly sequence contained 13 high confidence genes annotated in the IWGSC v1.0 genome release (**Table 3**). A gene containing a putative LRR motif (TraesCS4B01G301300) could be the likely candidate for *Lr49*. However, despite having good sequence read coverage in both VL404 and WL711, no nucleotide variation was identified in the coding sequence of the gene that would result in an asynonymous mutation or premature stop codon. This finding suggests that this gene is an unlikely candidate for *Lr49*, although differences in gene expression caused by a noncoding variant between the parent lines cannot be ruled out.

**Table 3:** High confidence genes annotated in the IWGSC v1.0 genome sequence between flanking markers *sunKASP_21* and markers cosegregating with *sunKASP_24*.

IWGSC v1.0 Gene ID	IWGSC v1.0 human readable gene annotation
TraesCS4B01G300700	Carotenoid cleavage dioxygenase
TraesCS4B01G300800	Tryptophan synthase beta chain
TraesCS4B01G300900	Peptide chain release factor 1
TraesCS4B01G301000	DNA-directed RNA polymerase subunit beta
TraesCS4B01G301100	Receptor-like protein kinase
TraesCS4B01G301200	Hexosyltransferase
TraesCS4B01G301300	Leucine-rich repeat receptor-like protein kinase
TraesCS4B01G301400	B3 domain-containing protein
TraesCS4B01G301500	NAD(P)-binding Rossmann-fold superfamily protein
TraesCS4B01G301600	Origin recognition complex subunit 2
TraesCS4B01G301700	Transmembrane protein, putative
TraesCS4B01G301800	Alpha/beta-Hydrolases superfamily protein
TraesCS4B01G301900	Thionin-like protein
TraesCS4B01G302000	Agmatine coumaroyltransferase-2
TraesCS4B01G302100	Agmatine coumaroyltransferase-2
TraesCS4B01G302200	Agmatine coumaroyltransferase-2
TraesCS4B01G302300	Agmatine coumaroyltransferase-2
TraesCS4B01G302400	Uroporphyrinogen III synthase
TraesCS4B01G302500	Transmembrane protein 131
TraesCS4B01G302600	MADS box transcription factor
TraesCS4B01G302700	Vacuolar-sorting-associated protein 37-like protein
TraesCS4B01G302800	Aspartic proteinase nepenthesin-1
TraesCS4B01G302900	DNA-directed RNA polymerase subunit
TraesCS4B01G303000	Dentin sialophosphoprotein-related, putative isoform 1
TraesCS4B01G303100	WD and tetratricopeptide repeat protein, putative
TraesCS4B01G303200	Zinc finger protein
TraesCS4B01G303300	Ubiquitin carboxyl-terminal hydrolase 2

**Figure 4:**
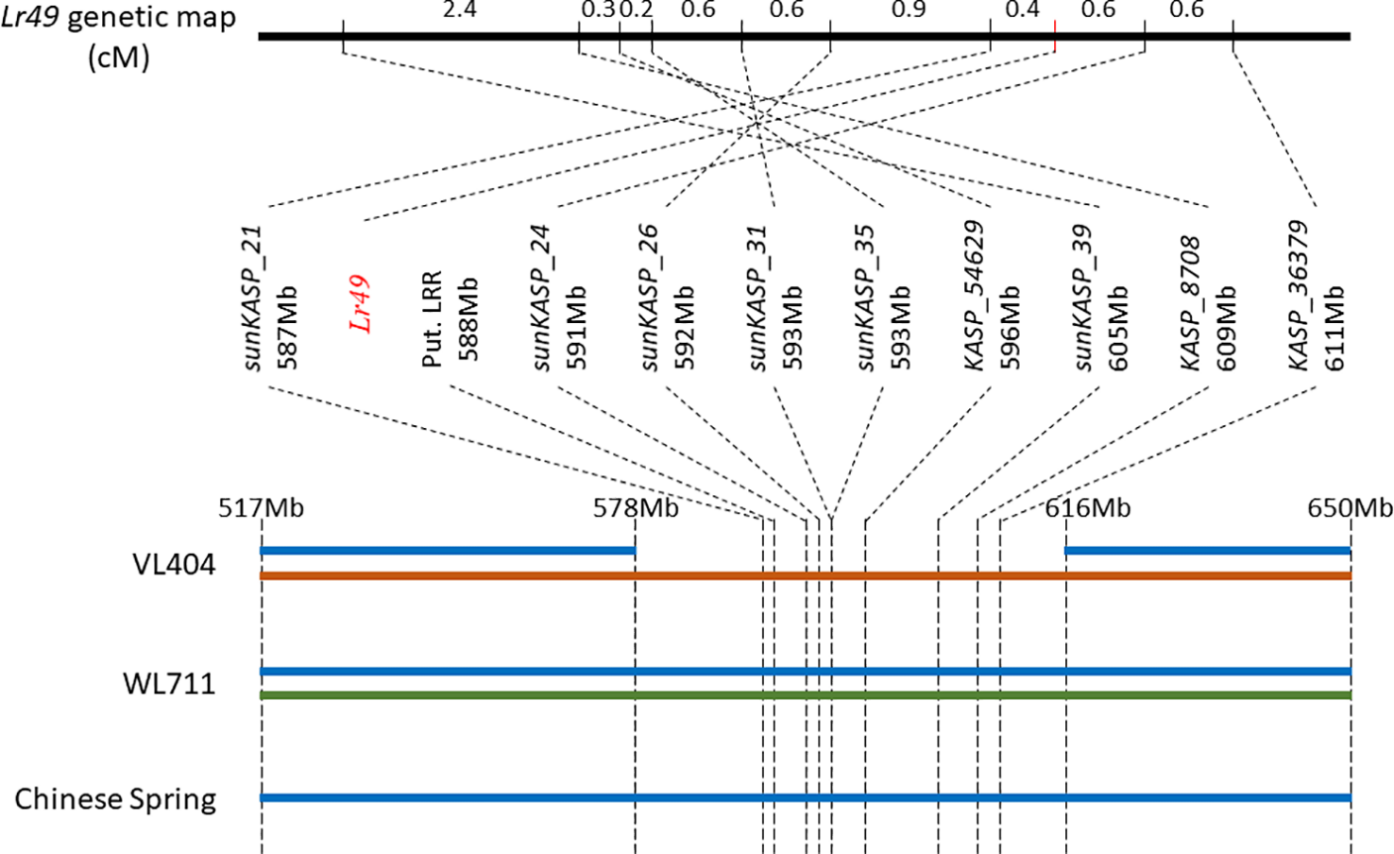
Diagrammatic representation of the *Lr49* interval and flanking region on chromosome 4B. Both VL404 (resistant parent) and WL711 (susceptible parent) have two sequence haplotypes outside the *Lr49* region, while VL404 has only one sequence haplotype within the *Lr49* interval. Chinese Spring has only one sequence haplotype across entire region. Each unique sequence haplotype is represented by a different color. Markers mapping within the *Lr49* interval are ordered based on their physical mapping location. The *Lr49* genetic map does not reflect the physical mapping order as illustrated by comparison of genetic and physical map order across the *Lr49* interval. Differences in sequence haplotype structure and physical-genetic map order in VL404 and WL711, relative to Chinese Spring, suggests structural rearrangement or copy number variation (CNV) relative to Chinese Spring.

The alignment of the flow-sorted chromosome paired-end sequence reads from each parent to the reference genome assembly of Chinese Spring using GYDLE software revealed the presence of two distinct sequence haplotypes in the susceptible parent WL711 that spanned the entire *Lr49* region delineated by markers *sunKASP_21* and *sunKASP_36379*. Indeed, these sequence haplotypes extended well beyond the *Lr49* region both distally and proximally (**Figure 4**). In contrast, a single but different sequence haplotype was observed in the resistant parent VL404 within the *Lr49* region delineated by markers *sunKASP_21* and *sunKASP_36379*. Outside this region, VL404 possessed two distinct sequence haplotypes, one of which was identical to one of those observed in WL711 and the single sequence haplotype present in Chinese Spring, which was observed when Chinese Spring flow-sorted chromosome paired-end sequence reads were aligned to pseudomolecule 4B (IWGSCv1.0) (**Figure 4**). The presence of only a single sequence haplotype within the *Lr49* region delineated by markers *sunKASP_21* and *sunKASP_36379* in VL404, relative to WL711, was further supported by paired-end sequence read coverage across the interval (**Supplementary Figure 2**). These observations indicate the presence of structural chromosomal differences between VL404, WL711 and Chinese Spring.

## Discussion

Cultivars carrying long-lasting resistance to diseases have been released through conventional phenotypic screening; however, the genetic basis of resistance has been largely unknown. It is challenging to combine ASR and APR genes with confidence into a single genotype using phenotypic assays, as the high level of resistance conditioned by effective ASR genes masks the detection of APR loci. Molecular markers linked with rust resistance genes developed through fine mapping can be used to pyramid resistance genes into a single genotype efficiently and reliably.

We used high-density genotyping and flow-sorted chromosome sequencing to fine-map the genomic region on chromosome 4B containing *Lr49* as a first step towards developing a diagnostic marker for use in marker assisted selection and for cloning of this gene. Genetic mapping of VL404/WL711 RIL population using the iSelect 90K wheat SNP bead chip array localized *Lr49* to a 3.3-cM interval. Subsequent mapping using KASP markers targeting nucleotide variation identified from flow-sorted chromosome sequences of the parental lines VL404 and WL711 that was expected to tile across the physical region corresponding to *Lr49* in the Chinese Spring chromosome 4B pseudomolecule, further reduced the *Lr49* interval to 1.0 cM (**Figure 1**).

While the availability of flow-sorted chromosome sequences for the parental lines VL404 and WL711 allowed the rapid identification of nucleotide variation to further fine map the *Lr49* interval and to largely preclude a high-confidence gene containing a LRR motif as a candidate for *Lr49*, many of the KASP markers targeting this polymorphism could not be reliably scored in the mapping population. Indeed only seven of 28 KASP assays developed for this purpose could be genetically mapped. Comparison of the *Lr49* genetic linkage map with the Chinese Spring chromosome 4B physical map (**Figure 3**), followed by visualization of the alignment of the flow-sorted chromosome paired-end sequence reads from each parent to the reference genome assembly of Chinese Spring (**Figure 4**), suggested structural variations in VL404 and WL711 compared to Chinese Spring. This was supported by observed collinearity of high confidence genes across the orthologous region in *Ae. tauschii* chromosome 4D, and homoeologous regions in Chinese Spring chromosome 4D, emmer chromosome 4B, and Chinese Spring chromosome 4B (**Supplementary Figure 1**). These observations suggested that the linkage map appeared inverted and overlapping when compared to the physical position of the markers in the Chinese Spring 4B pseudomolecule (**Figure 3**), and the presence of two sequence haplotypes in WL711 across the *Lr49* region and beyond the *Lr49* interval in VL404 (**Figure 4**). These results implied the presence of an inverted local duplication in each of the parental lines relative to Chinese Spring. The presence of two distinct sequence haplotypes across the *Lr49* region in the susceptible parent W711 and only one in the resistant parent VL404 suggested further localized structural variation between the parental lines (**Figure 4**). This structural variation is likely to explain the difficulty encountered for developing scorable KASP markers for polymorphism identified from the parental flow sorted chromosome sequence. KASP markers that assay multiple loci exhibit cluster compression due to the increase in allele dosage, and therefore are more difficult to score compared to a KASP marker that assays only a single locus. Cluster compression is expected for KASP markers that assay a duplicated genomic region. The presence of extensive local structural variation between wheat cultivars has been previously reported (Montenegro et al., 2017; Clavijo et al., 2017).

It is unclear whether the presence of only one haplotype for the *Lr49* region in VL404 represents a structural deletion, or the presence of a chromosomal segment that is sufficiently diverged at the nucleotide level such that paired-end reads from this region in VL404 could not be aligned to the assembled genome sequence of Chinese Spring. As Chinese Spring does not carry *Lr49*, it is likely that VL404 carries a resistance gene absent in Chinese Spring. This scenario is possible since VL404 was derived through crosses involving durum wheat. We are currently using various sequencing technologies including Nextera mate-pair sequencing and Dovetail Genomics scaffolding technology (Thind et al., 2017) to *de novo* assemble the *Lr49* region for VL404 and WL711, which will elucidate the physical structure and help to clone *Lr49* and develop a diagnostic marker for use in breeding.

Marker *sunKASP_21* showed the strongest association with *Lr49* when tested on a diverse set of 75 Australian and 90 European cultivars, which were unlikely to carry *Lr49*. The marker amplified the VL404 allele in only three genotypes (Gazelle, JO 8023 and Safir) and the non-*Lr49* associated alleles in the remaining 162 genotypes. This result was supported by the major SNP haplotypes for the *Lr49* region revealed by the 890 globally diverse exome sequenced accessions, which showed evidence for two historical recombination sites distal to *sunKASP_21* (**Figure 2**). The combination of these two pieces of evidence suggests that *sunKASP_21* can be used for marker assisted deployment of *Lr49* in breeding programs.

In conclusion, our study has demonstrated application of the assembled reference genome sequence for cultivar Chinese Spring and the flow-sorted chromosome sequences of the parental lines to accelerate fine-mapping of trait loci in common wheat. Our results also highlight the challenge for cloning *Lr49* caused by the presence of structural variation in the parental lines of the mapping population, relative to Chinese Spring, and the importance of being able to assemble such regions to enable the cloning of causal genes and development of diagnostic markers for use in breeding.

## Data Availability Statement

The SNP data used in the study were previously generated and can be found in the European Nucleotide Archive using accession number ERZ805275 (https://www.ebi.ac.uk/ena/data/view/ERZ805275).

## Author Contributions

VN conducted initial mapping of KASP markers and drafted the manuscript. DB developed high resolution population and tested flanking markers on it. MV and JD sorted chromosome 4B from parental lines and conducted sequencing. NQ mapped markers developed from the flow sorted chromosomes. PK, KF, and MH aligned flow sorted chromosome sequences with the reference sequence. UB developed KASP and *sunKASP* markers. PK, MH, UB, and HB edited the manuscript. UB and HB provided overall supervision. All authors read the manuscript.

## Funding

VN was funded by Australia Awards and the Ugandan Government. UB, HB, PK, KF and MH were funded through research grants from the Grains Research & Development Corporation (GRDC) Australia. MV and JD were supported by the ERDF project “Plants as a tool for sustainable global development” (No. CZ.02.1.01/0.0/0.0/16_019/0000827).

## Conflict of Interest

The authors declare that the research was conducted in the absence of any commercial or financial relationships that could be construed as a potential conflict of interest.
