# Supplementary material for: Fine Mapping of Lr49 Using 90K SNP Chip Array and Flow-Sorted Chromosome Sequencing in Wheat
Source: Front Plant Sci. 2020 Feb 4;10:1787. doi: 10.3389/fpls.2019.01787 (PMC7010802; doi:10.3389/fpls.2019.01787)
Supplement: Supplementary file 1 [file Image_1.pdf]

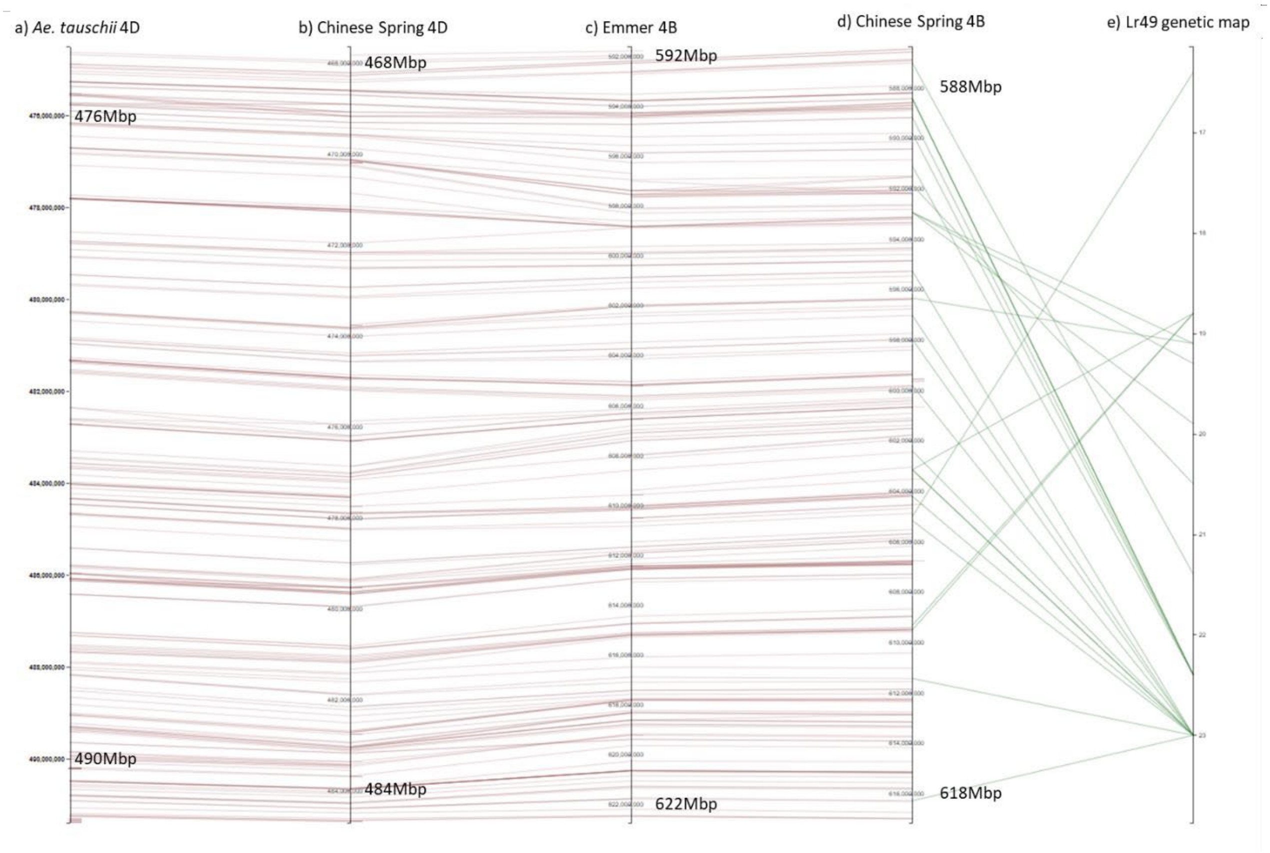

Supplementary Figure 1. Pretzel (http://plantinformatics.io) visualisation of synteny between (a) *Ae. tauschii* chromosome 4D, (b) Chinese Spring chromosome 4D, (c) emmer chromosome 4B, (d) Chinese Spring chromosome 4B, and (e) the *Lr49* genetic map, based on physical mapping of high confidence genes across the *Lr49* region (a to d) and corresponding physical map position (d) and genetic map position (e) of markers across the *Lr49* region. Red lines link high confidence genes. Green lines link markers. High confidence genes in all chromosomes are colinear, while the genetic map order of markers across the *Lr49* region show rearrangement relative to their physical mapping order in Chinese Spring.
