# Supplementary material for: Fine Mapping of Lr49 Using 90K SNP Chip Array and Flow-Sorted Chromosome Sequencing in Wheat
Source: Front Plant Sci. 2020 Feb 4;10:1787. doi: 10.3389/fpls.2019.01787 (PMC7010802; doi:10.3389/fpls.2019.01787)
Supplement: Supplementary file 2 [file Image_2.pdf]

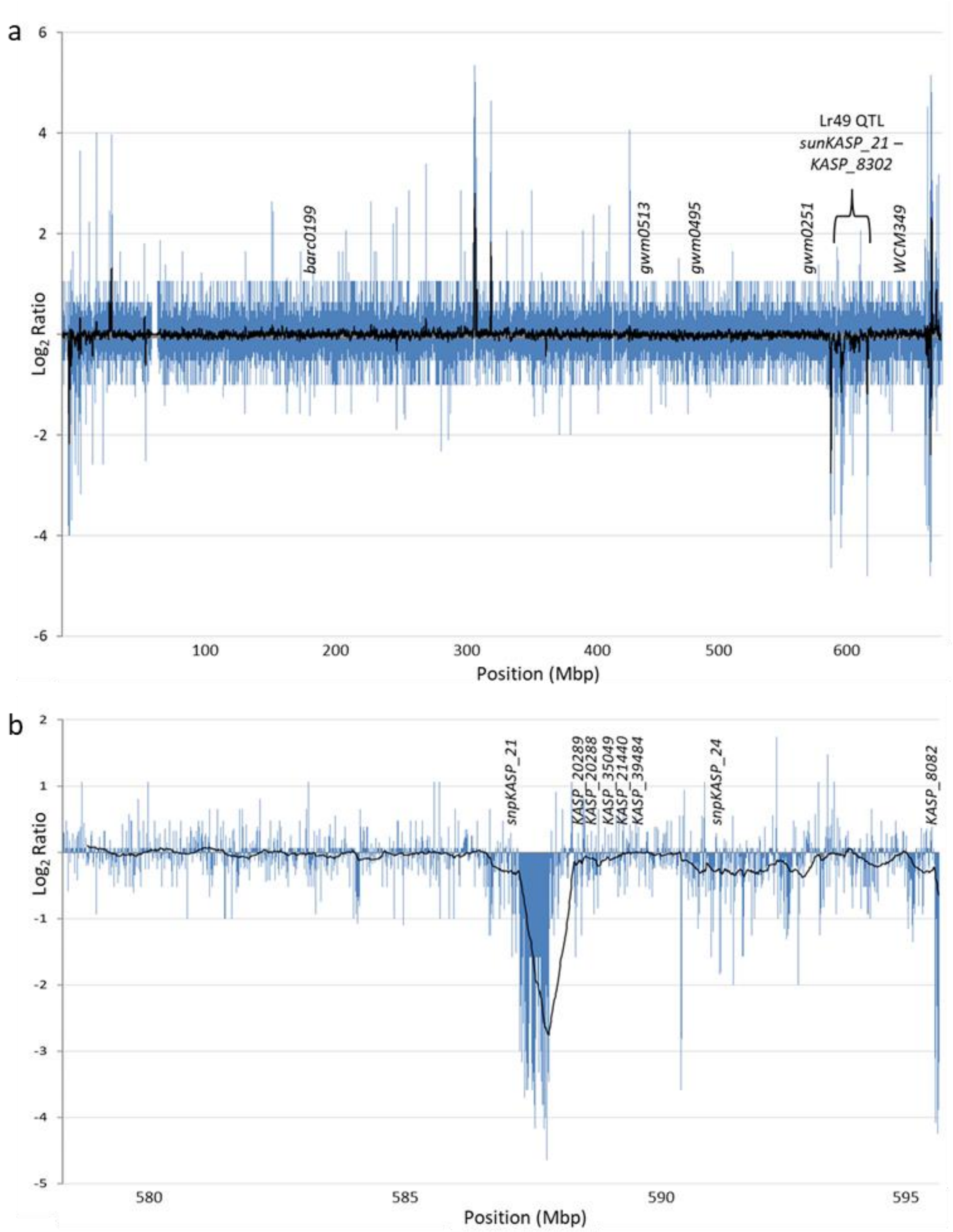

Supplementary Figure 2. Log_2 ratio plot (VL404:WL711) of average paired-end sequence read coverage across (a) chromosome 4B, and (b) *Lr49* region. Blue and black lines represent sequence coverage based on 10 and 500 kb sliding windows, respectively. Physical mapping position of microsatellite markers and the *Lr49* interval in chromosome 4B are shown in (a). The position of the putative deletion in VL404, relative to WL711, along with physical mapping position for markers that validated (*sunKASP_21* and *KASP_8082*) and did not validate (*KASP_20289*, *KASP_20288*, *KASP_35049*, *KASP_21440*, *KASP_39484* and *sunKASP_24*) are shown in (b).
